# Selection of Recipient Vessels in Double-Barrel STA-MCA Bypass Surgery with the Assistance of Intraoperative ICG Fluorescence: A Case Report and Review of the Literature

**DOI:** 10.3390/brainsci16030316

**Published:** 2026-03-16

**Authors:** Stefanie Bauer, Timo Kahles, Michael Diepers, Gerrit A. Schubert, Lukas Andereggen, Serge Marbacher

**Affiliations:** 1 Department of Neurosurgery, Kantonsspital Aarau, 5001 Aarau, Switzerland; 2 Department of Neurology, Kantonsspital Aarau, 5001 Aarau, Switzerland; 3 Institute of Neuroradiology, Department of Radiology, Kantonsspital Aarau, 5001 Aarau, Switzerland; 4 Department of Neurosurgery, RWTH Aachen University Hospital Aachen, 52074 Aachen, Germany; 5 Faculty of Medicine, University of Bern, 3012 Bern, Switzerland

**Keywords:** STA-MCA EC/IC bypass, double barrel bypass, ICG-VA, chronic cerebral ischemia, steno-occlusive arterial disease, recipient vessel identification

## Abstract

**Highlights:**

**What are the main findings?**

- Intraoperative indocyanine green video angiography (ICG-VA) visualizes relative cortical perfusion delay in steno-occlusive disease during STA-MCA bypass surgery.
- This case report demonstrates the application of ICG-VA for recipient vessel selection in double-barrel STA-MCA bypass.

**What are the implications of the main findings?**

- Perfusion-oriented assessment may complement conventional anatomical criteria during flow-augmentation bypass.
- Qualitative real-time fluorescence imaging may support intraoperative decision-making without additional invasive imaging.

**Abstract:**

Background/Objectives: Selection of the optimal recipient artery in superficial temporal artery to middle cerebral artery (STA–MCA) extracranial–intracranial bypass surgery is essential to ensure adequate cerebral perfusion. Various pre- and intraoperative tools for target vessel selection have been proposed. Indocyanine green fluorescence video angiography (ICG-VA) enables real-time visualization of cerebral hemodynamics, facilitating recipient vessel selection and anastomotic evaluation. Here, we review the literature and present the use of qualitative ICG-VA to support intraoperative decision-making during double-barrel (DB) STA–MCA bypass surgery. Case description: We report the case of a 68-year-old patient with bilateral steno-occlusive cerebrovascular disease, who developed progressive hemodynamic compromise of the left hemisphere after prior right-sided STA-MCA bypass. Preoperative imaging demonstrated impaired perfusion and posterior-to-anterior leptomeningeal collateralization from the posterior cerebral artery. During the left-sided DB bypass surgery, intravenous ICG-VA was used to assess relative cortical perfusion. Two superficial M4 branches with the most pronounced perfusion delay were selected as recipients based on the ICG-VA and anatomical criteria. Postoperative angiography confirmed graft patency. At short-term follow-up, the patient remained neurologically stable, with complete regression of preoperative symptoms. Conclusions: This case illustrates the application of qualitative ICG-VA for perfusion-oriented recipient vessel selection in DB STA-MCA bypass for steno-occlusive disease. Real-time perfusion assessment may complement conventional anatomical criteria for recipient vessel selection in flow-augmentation procedures. Further studies incorporating quantitative hemodynamic analysis are warranted.

## 1. Introduction

In patients with steno-occlusive cerebrovascular disease who present with recurrent ischemic symptoms and impaired cerebrovascular reserve (hemodynamic failure type II), STA-MCA bypass surgery may be considered when medical therapy is insufficient [1,2]. Although the COSS trial did not demonstrate an overall reduction in ipsilateral stroke risk [3], surgical revascularization remains a therapeutic option in carefully selected patients with documented hemodynamic compromise, where cerebral blood flow augmentation may be beneficial [1,4,5]. In the past decade, the DB STA-MCA bypass has emerged. This bypass may offer advantages in patients with steno-occlusive disease and limited collateral capacity, especially when perfusion deficits involve broader cortical regions requiring augmented flow [6]. Cherian et al. showed that DB STA-MCA bypasses may be considered ‘high-flow’ bypasses with relatively large flow capacities of 30–120 cc/min [7]. Nonetheless, the success of any cerebral bypass depends on selecting the right recipient vessel. Several tools can be used during the surgery such as navigation guidance through preoperative imaging, intraoperative Doppler sonography, intraoperative digital subtraction angiography (DSA) or intraoperative ICG-VA [8]. We review the literature and present a case using ICG-VA to select the optimal recipient vessels during DB STA-MCA bypass surgery.

## 2. Case Description

**Figure 1:**
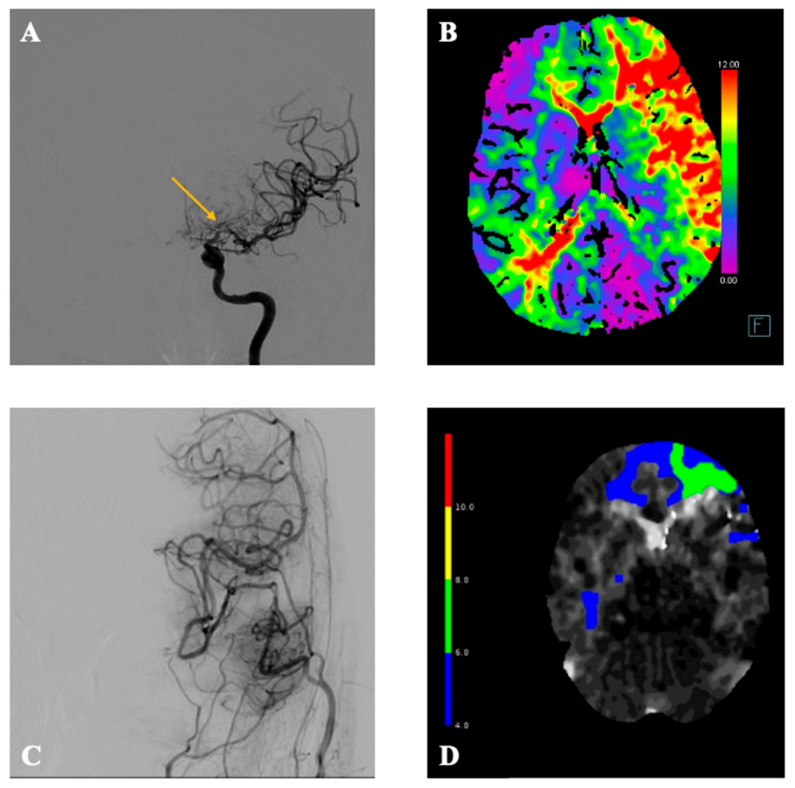
(**A**,**B**) show the preoperative digital subtraction angiography and the CT perfusion scan with time-to-maximum scan (Tmax). The yellow arrow in (**A**) points towards the stenosis of the M1 branch on the left side. Perfusion imaging (**B**) demonstrates delayed perfusion of the entire left MCA territory with a scale from 4 to 12 s. (**C**,**D**) show the imaging studies 2 months postoperatively with the digital subtraction angiography on the left side (**C**). The postoperative perfusion CT with scale from 4 to 12 s demonstrates largely restored perfusion after the DB STA-MCA bypass with a delayed perfusion frontal left (**D**).

A 68-year-old male patient initially presented with a history of recurrent ischemic stroke due to atherosclerotic right-sided M1 occlusion of the MCA. The patient was successfully treated with a traditional DB STA-MCA bypass on the right side three years ago. During follow-up, an occlusion of the contralateral M1-branch occurred with subsequent hemodynamic failure type II. Clinically, the patient presented with blood pressure-dependent right-sided faciobrachial hemiparesis. The CT-angiogram (CTA) visualized a large perfusion mismatch on the left hemisphere due to progression of the already known stenosis of the M1 branch on the left side with decompensation of the collateral vessels (Figure 1). The preoperative DSA with selective vertebral artery injection revealed leptomeningeal collateralization from the posterior cerebral artery (PCA) to distal MCA branches, particularly supplying the temporal cortical region. This retrograde filling pattern reflected compensatory pial anastomoses due to proximal M1 occlusion. The patient was discussed at our interdisciplinary neurovascular board, where the treatment with a DB STA-MCA bypass on the left side was indicated.

**Figure 2:**
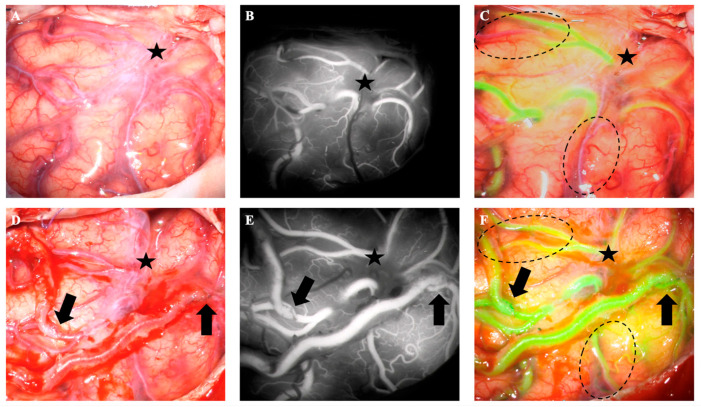
Pictures (**A**–**C**) show the baseline native, the ICG and ICG-VA. The black star in pictures (**A**–**F**) denotes the Sylvian fissure. Pictures (**D**–**F**) show the native and the ICG-VA after anastomosis of the temporal and frontal bypass. The downward pointing black arrow points toward the temporal bypass anastomosis site and the upward pointing black arrow toward the frontal anastomosis site. ICG-VA shows patency of both bypasses. The dashed line in (**C**,**F**) shows two other possible recipient vessels. In (**C**) delayed perfusion of the other possible recipient vessels is seen on ICG-VA; after bypass to other recipient vessels, both vessels fill up over retrograde flow without perfusion delay (**F**).

Intraoperatively, we used ICG-VA to guide decision-making in choosing the most promising recipient arteries. After dural opening, 25 mg ICG was applied via peripheral venous line. We used a ZEISSS KINEVO 900 microscope (Carl Zeiss Meditec AG, Jena, Germany) with the integrated INFRARED 800 module to visualize the fluorescence. Several cortical MCA branches demonstrated delayed fluorescence arrival compared to adjacent vessels. Two superficial cortical branches on the frontal and temporal convexity exhibited the most pronounced perfusion delay. Recipient selection was based on the relative fluorescence delay suggesting hemodynamic compromise, sufficient vessel diameter (≥1 mm) for safe anastomosis, and favorable accessibility within the operative field (Figure 2). After anastomosis of both vessels to the STA branches, another ICG-VA was performed to evaluate the patency of both bypasses (Figure 2). The patient recovered quickly after surgery with complete regression of symptoms.

**Figure 3:**
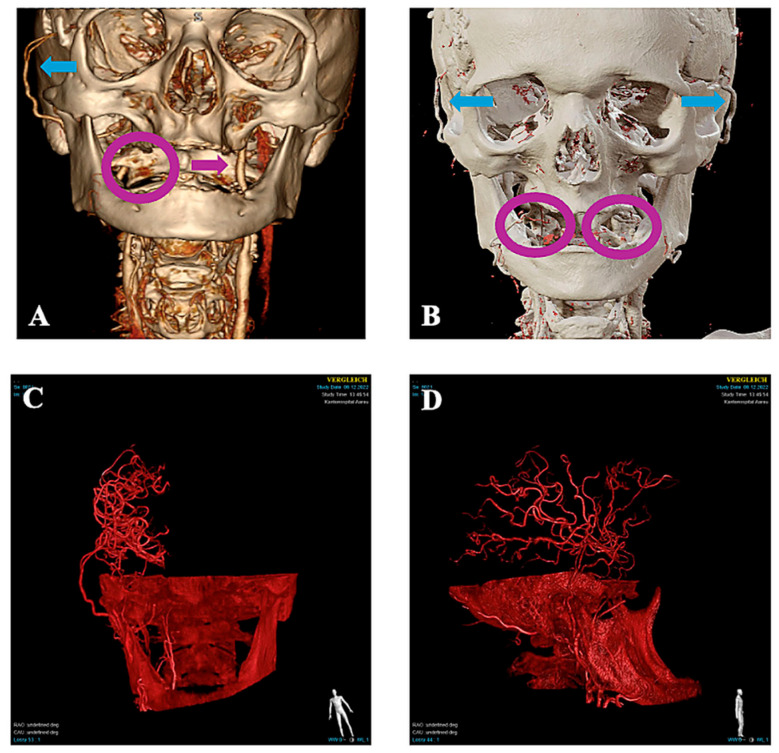
Three-dimensional postoperative CT angiography reconstructions after STA-MCA bypass surgery. (**A**) Reconstruction after right-sided DB STA-MCA bypass surgery. (**B**) Reconstruction after the second surgery, demonstrating DB STA-MCA bypasses on both sides. Blue arrows indicate the STA-MCA bypasses in (**A**,**B**). In (**A**), the purple circle marks the expected location of the right internal carotid artery, which is not visualized on the 3D CTA reconstruction, while the purple arrow indicates the left internal carotid artery. In (**B**), the purple circles mark the expected locations of both internal carotid arteries, which are not visualized on the 3D CTA reconstruction, consistent with severe steno-occlusive disease. The bottom row shows the perfusion territories of all four bypasses: (**C**) right DB STA-MCA bypass and (**D**) left DB STA-MCA bypass.

At the follow-up visit 2 months postoperatively, the patient demonstrated sustained clinical stability, reporting feeling higher energy levels than before surgery and with no new neurological deficits. Although CTA and CTP at 2 months raised suspicion of possible compromise of the left frontal bypass due to focal perfusion delay, a confirmatory DSA demonstrated the patency of all four bypass grafts (Figure 1 and Figure 3), and MRI excluded new ischemia. Serial neurovascular ultrasound examinations at 1 and 5 months postoperatively likewise demonstrated the sustained patency of both STA donor branches. The patient is still closely monitored in the neurologic outpatient clinic with regular clinical checks and neurovascular ultrasound.

## 3. Discussion

Selecting the right recipient vessel is crucial to achieving optimal revascularization in cerebral bypass surgery. Traditional recipient vessel selection relies on preoperative imaging, anatomical landmarks, and intraoperative vessel appearance upon visual inspection and Doppler sonography. For the STA-MCA bypass, the recipient vessel should have a diameter similar to the diameter of the STA [2]. Some tools have emerged in the past that help in recipient vessel selection. Some surgeons may use intraoperative doppler sonography to evaluate the blood flow of potential recipients. However, doppler signal interpretation is difficult and remains subjective for the interpreter [9]. Intraoperative digital subtraction angiography may not only be used to evaluate bypass patency but also to support the surgeon in recipient vessel identification and evaluation of perfusion of cortical vessels. But its routine use in cerebral bypass surgery is limited by its invasiveness, high costs and increased duration of surgery [8]. ICG-VA and its quantitative analysis with, e.g., FLOW800 (Carl Zeiss Meditec AG, Jena, Germany) are established tools in cerebrovascular surgery and have long been used to assess the patency of anastomoses [10,11]. Lawton and colleagues proposed the technique called ‘flash fluorescence’ in 2011, which is a convenient method to identify recipient vessels in aneurysmal bypass surgery (Table 1). Hereby, ICG is applied after temporary clipping of the necessary artery showing perfusion delay in some cortical arteries. If these arteries fill rapidly with ICG after removal of the temporary clips, they are deemed suitable as recipient vessels [12]. Furthermore, ICG-VA emerged to be useful in refining recipient vessel selection. ICG-VA is readily available and provides real-time perfusion data, thus facilitating the identification of vessels with delayed perfusion [13]. In 2012, Esposito et al. defined a primary and secondary identification of recipient vessels using ICG-VA in cerebral bypass surgery for the treatment of complex MCA aneurysms. In primary identification, the baseline ICG-VA already showed a delayed filling of a cortical artery due to increased resistance to flow. In secondary identification, the involved cortical arteries were identified with a flow delay after temporary occlusion of an MCA branch [13].

In cases of steno-occlusive disease, the baseline ICG-VA already shows delayed vessel filling. Thus, the concept of primary identification from Esposito et al. [13]. may be used. In our case, we identified a frontal and a temporal cortical (M4) branch of the left MCA with delayed filling upon ICG-VA. Both these branches had a diameter >1 mm and were easily accessible in the operating field, allowing a structured selection strategy that combined perfusion-based assessment with conventional anatomical criteria. Postoperative imaging studies revealed the restored perfusion of the entire left hemisphere, and the neurologic symptoms fully regressed—supporting the adequacy of the selected recipient vessels.

In routine surgical practice, sequential intraoperative ICG-VA assessment may further refine hemodynamic evaluation during bypass procedures. Performing ICG-VA at baseline, after completion of the first anastomosis, and again after the second bypass can help assess the degree of perfusion improvement following each step of revascularization. If cortical perfusion improves sufficiently after the first anastomosis, performing a second anastomosis may be unwarranted and could theoretically increase the risk of postoperative hyperperfusion syndrome. Intraoperative perfusion assessment using ICG-VA and its quantitative analysis have been shown to detect bypass-related hemodynamic changes and may help identify patients at risk for hyperperfusion [14,15]. In the present case, the decision to perform a double-barrel bypass had already been established preoperatively, based on the CT perfusion imaging demonstrating pronounced hemispheric hypoperfusion and insufficient collateralization, suggesting that more extensive flow augmentation would be required.

Nevertheless, the interpretation of ICG-VA remains qualitative, as quantitative intraoperative flow measurements such as FLOW800-based fluorescence analysis were not performed in this case. Consequently, the objective assessment of flow volume or pressure gradients across the anastomoses was not available. In addition, longitudinal quantitative perfusion imaging beyond the early postoperative phase was limited. While clinical and neurovascular ultrasound confirmed patency at 5 months postoperatively, the degree of long-term hemodynamic augmentation cannot be precisely quantified.

The concept of intraoperative selection of the optimal recipient vessel in a patient with ischemic cerebrovascular disease was first described by Matsumoto in 2018 [16]. Recently, a larger case series was published describing the use of ICG-VA for intraoperative vessel-selection in patients with moyamoya disease and atherosclerotic cerebrovascular disease [17]. However, all patients in this study underwent single barrel (SB) bypasses. We presented here the first case of DB STA-MCA bypass for steno-occlusive disease using intraoperative ICG-VA for recipient vessel selection and confirmed its usefulness.

**Table 1:** All case reports or case series found in Pubmed/MEDLINE that report on cerebral bypass surgery and the use of ICG-VA for recipient vessel identification. SB single-barrel, DB double-barrel. MMV moyamoya vasculopathy. ACVD atherosclerotic cerebrovascular disease.

Author	Year	Number of Cases	SB or DB	Indication for EC-IC Bypass	Tools for Recipient Vessel Identification
Rodríguez-Hernández [12]	2011	3	3 SB	Aneurysm surgery (100%)	ICG-VA (‘Flash-Fluorescence’)
Esposito [18]	2012	7	5 SB, 2 DB	Aneurysm surgery (100%)	ICG-VA (‘primary’ and ‘secondary’ identification)
Esposito [19]	2014	2	SB	Aneurysm surgery (100%)	ICG-VA (‘primary’ and ‘secondary’ identification)
Matsumoto [16]	2018	1	SB	Acute cerebral ischemia	ICG-VA (delayed filling)
Goldberg [17]	2021	60	SB	MMV (68%), ACVD (32%)	ICG-VA, FLOW800
Current Case	2025	1	DB	Steno-occlusive disease (bilateral M1 stenosis)	ICG-VA (delayed filling)

## 4. Conclusions

This case report demonstrates that ICG-VA can assist in recipient vessel selection during DB STA-MCA bypass surgery in patients with steno-occlusive cerebrovascular disease. The real-time visualization of delayed cortical perfusion supported structured vessel selection and was associated with favorable short-term clinical and radiological outcomes. However, larger prospective studies incorporating quantitative hemodynamic analysis are required to further evaluate the routine use of ICG-VA for recipient vessel selection in EC-IC bypass procedures.

## Data Availability

The original contributions presented in the study are included in the article. Additional inquiries can be directed to the corresponding authors.

## Abbreviations

The following abbreviations are used in this manuscript:

STA-MCA	Superficial temporal artery–medial cerebral artery
EC-IC	Extracranial–intracranial
SB	Single barrel
DB	Double barrel
ICG-VA	Indocyanine green video angiography
CT	Computed tomography
CTA	Computed tomography angiography
DSA	Digital subtraction angiography
Tmax	Time-to-Maximum
